# Joint Trajectories of Lifestyle Indicators and Their Associations with Blood Pressure among Chinese Middle School Students

**DOI:** 10.3390/nu16172994

**Published:** 2024-09-05

**Authors:** Guangzhuang Jing, Xinxin Liu, Jiaojiao Shi, Junlei Xue, Hui Peng, Huijing Shi

**Affiliations:** 1 Department of Maternal, Child and Adolescent Health, School of Public Health, Fudan University, Dong’an Road, 130, Shanghai 200032, China; 21111020064@m.fudan.edu.cn (G.J.); 22111020066@m.fudan.edu.cn (J.S.); 2 The Jiading District Center for Disease Control and Prevention, Tacheng Road, 264, Jiading District, Shanghai 201800, China; jdcdcxxws@163.com (X.L.); raychar@163.com (J.X.)

**Keywords:** lifestyle behaviors, dietary behavior, physical activity, screen time, sleep duration, longitudinal trajectory, blood pressure, middle school students

## Abstract

Lifestyle behaviors, defined as a combination of dietary behavior, physical activity (PA), screen time (ST), and sleep duration indicators, are strongly associated with blood pressure (BP) in students. Our aim was to characterize the joint trajectories of lifestyle behaviors among middle school students and evaluate their association with BP. Data were obtained from the monitoring dataset on common diseases and health factors among students in Jiading District, Shanghai, China, conducted from 2019 to 2023. Lifestyle behavior data were collected annually from middle school students aged 12–18 years through questionnaires covering dietary behavior score, PA, ST, and sleep duration. Students’ BP was measured in 2023. Joint trajectories of lifestyle behaviors were determined using group-based multi-trajectory modeling. Associations between lifestyle trajectories and students’ BP were examined using multiple linear regression and modified Poisson regression. A total of 1378 middle school students (759 [58.98%] boys, median age 14.36 years [IQR: 13·30–13.28]) with lifestyle behaviors data assessed at least three times were included, and they were categorized into four joint lifestyle trajectories as follows: “remain unhealthy with low PA and increasing ST” (n = 141, 10.46%), “remain unhealthy with only low PA” (n = 305, 22.63%), “change towards unhealthy with decreasing sleep duration” (n = 776, 57.57%), and “relatively healthy” (n = 126, 9.35%). After adjusting for important confounders, the “remain unhealthy with low PA and increasing ST” group was associated with higher diastolic BP (DBP) [β: 3.49, 95% CI: 0.55–6.44] and higher mean arterial pressure (MAP) [β: 3.19, 95% CI: 0.37–6.01] in students compared with the “relatively healthy” group. Additionally, compared with the “relatively healthy” group, students in the “remain unhealthy with low PA and increasing ST” group had a 1.12-fold increase in the risk of hypertension (risk ratios: 1.12, 95% CI: 1.03–1.24). All trend *p* values in DBP, MAP, and hypertension from the “relatively healthy” group to the “remain unhealthy with low PA and increasing ST” group were less than 0.05. Four distinct lifestyle trajectories were identified among middle school students. Students who remained in the “unhealthy with low PA and increasing ST” lifestyle trajectory were associated with later elevations in BP.

## 1. Introduction

Hypertension is a primary risk factor for cardiovascular disease and a major contributor to the global burden of disease [1,2]. Particularly, the increasing prevalence of childhood hypertension has become a major public health concern [3,4,5]. A systematic literature review indicated that the global prevalence of childhood hypertension was 4.00% (95% CI, 3.29–4.78%) until 2018 based on blood pressure (BP) measurements in at least three separate visits [4]. In China, the standardized prevalence of hypertension among children aged 12–17 years in 2015 was 8.8% [6]. Numerous clinical and epidemiological studies with long-term follow-up have established clear associations between elevated BP in childhood and the development of hypertension [7] and cardiovascular subclinical impairment [8,9] in adulthood. Consequently, identifying modifiable risk factors for childhood hypertension and implementing population-based interventions are critical strategies for preventing cardiovascular diseases.

The progression of hypertension in adolescents is often influenced by a complex interplay of multiple lifestyle behaviors that are measurable and interdependent. Previous studies have demonstrated that unhealthy lifestyle behaviors, such as unhealthy dietary behaviors (e.g., high consumption of sweetened beverages [10], fried foods [11], low intake of fruits and vegetables [12]), inadequate physical activity (PA) [13], insufficient sleep [14], and excessive screen time (ST) [15]), are modifiable factors that can contribute to the prevention of children hypertension. However, multiple behavioral patterns usually coexist within individuals, particularly in the context of rapid socioeconomic development in China. The increasing use of infrastructure such as mobile networks, screens, social media, and digital devices has significantly altered the behaviors of middle school students, resulting in many behaviors exhibiting clustering characteristics [16,17]. Therefore, exploring the evolution of multidimensional lifestyle behavior trajectories over time may provide valuable insights into the prevention of hypertension in children.

In recent years, an accumulating body of research has utilized group trajectory modeling to investigate children’s behavior [16,17,18]. The methodology purports to identify clusters of children who exhibit similar lifestyle trajectories across multiple related behaviors. However, the majority of the existing research concentrates on the association between individual lifestyle trajectories [19,20,21] and BP or sums multiple behaviors into a composite score [22,23], with limited exploration of how combined lifestyle behavior trajectories influence BP among middle school students. Therefore, it is essential to combine various behaviors to identify specific trajectories related to BP, enabling more targeted interventions.

To fill this gap, we aimed to identify the joint trajectories of lifestyle indicators by leveraging data from the 2019–2023 survey on common diseases and health factors among students in Jiading District, Shanghai, China, and examine their association with subsequent BP among middle school students. We hypothesized that specific combinations and patterns of lifestyle behaviors may exist among Chinese middle school students. Furthermore, certain combinations of these behaviors may be particularly associated with the occurrence and progression of BP.

## 2. Materials and Methods

### 2.1. Study Design and Participants

This study utilized monitoring data on common diseases and health factors among students in Jiading District, Shanghai. The dataset covered survey data obtained by the Jiading District Center for Disease Control and Prevention (Jiading CDC) from 2019 to 2023, involving elementary, middle, and high school students from six schools. Common disease monitoring indicators included height, weight, BP, myopia, dental examinations, scoliosis, and anemia. Health factor indicators included dietary behavior, PA, electronic screen use, outdoor activities, and sleep among primary and secondary school students.

**Figure 1:**
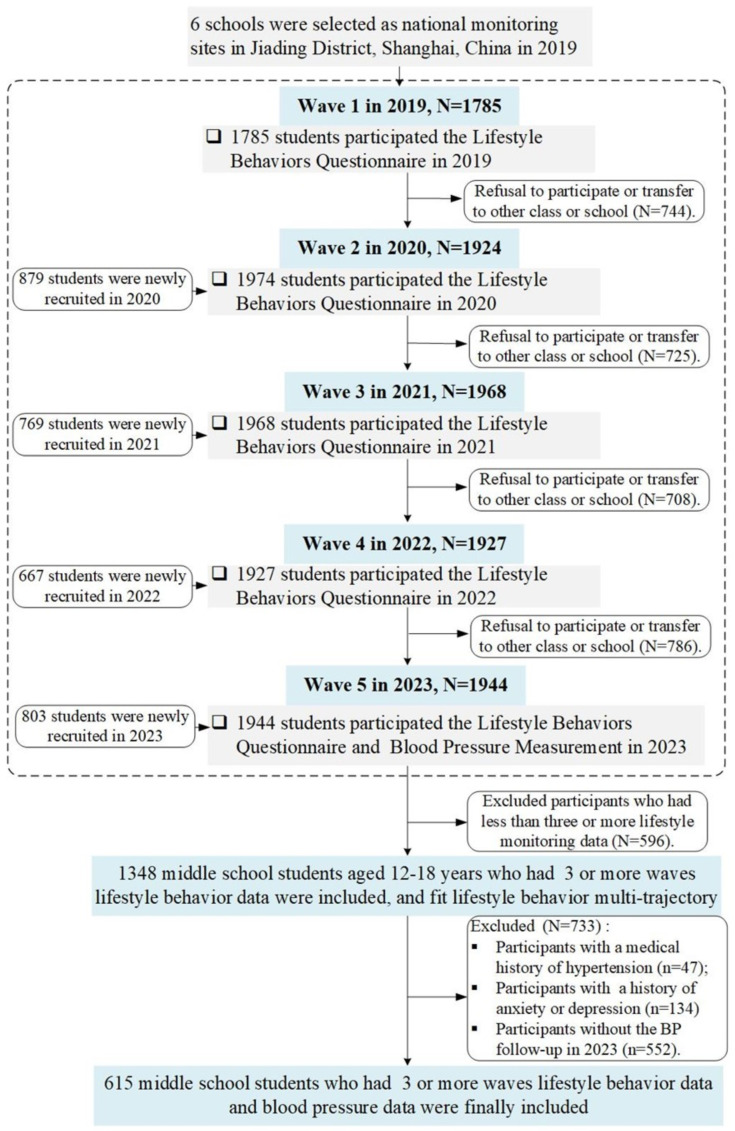
Flowchart of participants.

The Jiading CDC conducted annual monitoring of students separately in grades 3–6 in elementary school, grades 1–3 in middle school, and grades 1–3 in high school. Student tracking could be interrupted by graduation or school transfers. In this study, to ensure a high-quality fit for the trajectory model, we included 1348 middle school students aged 12–18 whose lifestyle behavior data assessed at least three waves. Subsequently, after excluding participants with a medical history of hypertension (n = 47), those with a history of anxiety or depression (n = 134), and those without the BP follow-up in 2023 (n = 671), the final sample size was 615. Figure 1 illustrates the inclusion and exclusion process for the annual sample sizes of students from 2019 to 2023.

Given that this study involves the follow-up investigation of common diseases and health factors among students from 2019 to 2023, it is considered a prospective observational study.

### 2.2. Lifestyle Behavior Measurement

According to a literature review published in *Hypertension* [24], the primary lifestyle behaviors influencing elevated BP in youth include overweight/obesity, diet, insufficient PA, excessive ST, and sleep disorders. Therefore, we selected the dietary behavior score, PA, ST, and sleep duration as indicators for trajectory analysis in this study, as these are the most relevant and modifiable factors among middle school students. The investigation of lifestyle behaviors was conducted using self-reported questionnaires originating from the “2019 national monitoring and intervention of common diseases and health influencing factors among students [25]”. The questionnaire demonstrated a Cronbach’s alpha coefficient of 0.90 and a content validity coefficient of 0.86.

#### 2.2.1. Dietary Behavior Score

The dietary behavior score was determined from dietary behaviors collected through self-reported questionnaires by the students. These included the following questionnaire items: a. the number of times sweetened beverages were consumed in the past seven days (e.g., cola, iced tea, orange juice with pulp, and nutri-express); b. the number of times fried foods were consumed in the past seven days (e.g., fried dough sticks, fried pancakes, French fries, and fried chicken wings); c. the number of times vegetables were consumed in the past seven days (both raw and cooked, such as salads, raw vegetables, or cooked vegetables); and d. the number of times fruits were consumed in the past seven days (excluding canned fruits).

The dietary behavior score was defined for each item based on the recommendations of the Dietary Guidelines for Chinese School-aged Children in 2022 [26] as follows: consuming sugary drinks fewer than once per day, consuming fried foods fewer than once per day, eating vegetables at least once per day, and eating fruits at least once per day. Each healthy behavior was assigned a score of 1, while unhealthy items were assigned a score of 0, resulting in a total score ranging from 0 to 4. This study assessed the total dietary behavior score for the four dietary behaviors of each student.

#### 2.2.2. PA

PA was assessed using a self-reported questionnaire that measured the number of days per week students engaged in moderate to vigorous PA for at least one hour. The specific questionnaire item was “For how many days in the past week did you engage in at least one hour of moderate to vigorous PA (can be accumulated)? Moderate to vigorous PA refer to exercises that make you breathe heavily or increase your heart rate, such as running, playing basketball, playing soccer, swimming, doing aerobics, or lifting heavy objects”.

#### 2.2.3. ST

ST was defined as the cumulative amount of time students used electronic screens on an average day in the past week. The specific questionnaire item was “In the past week, how much time on average per day did you spend watching TV, using a computer, mobile phone, game console, or other electronic screens?”

#### 2.2.4. Sleep Duration

Sleep duration was estimated based on the “students’ self-reported average sleep time on school days and weekends over the past week”. The specific questionnaire items were “On school days (Monday to Friday) over the past week, what was your average daily sleep time?” and “On weekends (Saturday and Sunday) over the past week, what was your average daily sleep time?”. The formula for calculating the average sleep duration was as follows: Average sleep duration = (average sleep duration on school days × 5 + average sleep duration on weekends × 2)/7.

### 2.3. Identifing Joint Trajectories of Lifestyle Behaviors

Group-based multi-trajectory modeling extends group-based univariate trajectory modeling to encompass multiple outcomes [27]. This method was used to identify latent clusters of children with similar lifestyle trajectories over time in four behaviors as follows: dietary behavior score, PA, ST, and sleep duration. All four lifestyle behaviors were treated as quantitative variables and fitted using a vector normal model. The optimal model classification was determined with the following criteria: a. the smallest absolute values of the Bayesian Information Criterion (BIC) and Akaike Information Criterion (AIC); b. an average posterior probability of greater than 0.8; c. a sample size of each group greater than 2% of the total participants; and d. an entropy value greater than 0.7. Finally, based on the above criteria, the analysis identified four combined trajectory groups, clustered in the age group of 13–15 years (model fit statistics are provided in Supplementary Table S1). The four trajectory groups were labeled according to their characteristics as follows: “remain unhealthy with low PA and increasing ST”, “remain unhealthy with only low PA”, “change towards unhealthy with decreasing sleep duration”, and the “relatively healthy” group. For detailed characteristics concerning trajectory groups, see the Results Section 3.1.

### 2.4. Students’ BP

Trained operators measured the students’ BP using an electronic sphygmomanometer (Omron HBP-1320) in accordance with the 2018 Chinese hypertension management guidelines [28]. BP was recorded twice at intervals of 1 to 2 min, and the average of these readings was noted. If the difference between the two systolic blood pressure (SBP) or diastolic blood pressure (DBP) readings exceeded 5 mm Hg, a third measurement was taken, and the average of the three readings was recorded. This study also focused on mean arterial pressure (MAP) alongside SBP and DBP, as MAP is an effective indicator of arterial load. MAP was calculated using the following formula: MAP = (SBP − DBP)/3 + DBP [29].

The students’ BP was evaluated using the 50th, 90th, 95th, and 99th percentile values based on gender, age, and height, following the reference of screening for elevated BP among children and adolescents aged 7~18 years (WS/T 610—2018) [30]. Hypertension was defined as SBP/DBP ≥ P_99_ + 5 mm Hg for students of the same sex, age, and height. High SBP was classified as SBP ≥ P_95_ for students of the same sex, age, and height, with normal DBP. Conversely, high DBP was defined as DBP ≥ P_95_ for students of the same sex, age, and height, with normal SBP.

### 2.5. Covariates

Covariates were determined based on previous studies [16,31,32,33], which were identified as relevant factors for lifestyle behaviors and BP in middle school students. Covariates included the children’s age at BP measurement, sex (boys/girls), body mass index (BMI) at BP measurement, self-perceived family income (poor/fine), and maternal education (junior high school or below/high school/college degree or above). Trained doctors measured the weight and height of the middle school students using an electronic scale. BMI was calculated by dividing the children’s weights (kg) by the square of their heights (cm). The sex, age, and self-perceived family income of the children and maternal education were assessed using self-reported questionnaires.

### 2.6. Statistical Analysis

A descriptive analysis was conducted on the lifestyle levels of middle school students from 2019 to 2023. The lifestyle data across five follow-up points were presented using the median (interquartile range [IQR]) and mean (standard deviation [SD]). Differences across the five data points were analyzed using analysis of variance (ANOVA) for normally distributed data and non-parametric tests for data that did not meet normal distribution criteria.

#### 2.6.1. Sociodemographic Characteristics across Trajectory Groups

The descriptive analysis of children’s sociodemographic characteristics was conducted according to four trajectory groups. Chi-square tests or ANOVA were used to compare participant sociodemographic characteristics. LSD and Bonferroni post hoc test methods were employed to assess comparisons between different trajectory groups and BP in students.

#### 2.6.2. Associations between Students’ Lifestyle Trajectories and Later BP

A multiple linear regression approach was used to estimate the β coefficient and 95% confidence interval (CI) for the relationship between the students’ lifestyle trajectories and subsequent BP values, adjusting for the covariates. Additionally, a modified Poisson regression with robust standard errors was employed to determine the risk ratios (RRs) of the students’ lifestyle trajectories for later BP outcomes (high SBP, high DBP, and hypertension). In all models, we adjusted the age, sex, BMI, and self-perceived family income of the children and maternal education. Considering that smoking and drinking alcohol may influence BP in students, a sensitivity analysis was conducted. Additional confounders for smoking and drinking alcohol in students were included in the original model, and the association between lifestyle behavior trajectories and BP was reanalyzed to verify the robustness of the primary analysis results. Smoking and drinking alcohol in students were assessed through self-reported questionnaires with the following items: smoking, “Have you ever smoked, even if it was just one or two puffs?”; drinking alcohol, “Have you ever consumed a whole glass of alcohol? (such as a can of beer, a shot of liquor, or a glass of wine)”.

All statistical analyses were performed using R version 4.1.2 and Stata 16.0 (StataCorp LLC, College Station, TX, USA), with a significance level of 0.05.

## 3. Results

Supplementary Table S2 presents the distribution characteristics of the lifestyle behavior indicators (dietary behavior score, PA, ST, and sleep duration) among middle school students from 2019 to 2023. Significant differences were observed in the distribution of these four lifestyle behaviors between 2019 and 2023 (*p* < 0.05).

Table 1 presents the baseline characteristics of 1378 middle school students, with a median age of 14.36 years (IQR: 13.30–17.28). More than half (58.98%) of the participants were boys. Approximately 7.48% of the students had hypertension. Additionally, more than half (62.46%) of the students reported poor self-perceived family income, and slightly more than half (57.42%) of the students reported that their mothers had a college degree or higher education level.

### 3.1. Characteristics of Lifestyle Trajectories

In the 1378 middle school students, group-based multi-trajectory modeling was used to identify the following four lifestyle trajectories: “remain unhealthy with low PA and increasing ST” (n = 141, 10.46%), “remain unhealthy with only low PA” (n = 305, 22.63%), “change towards unhealthy with decreasing sleep duration” (n = 776, 57.57%), and “relatively healthy” (n = 126, 9.35%), and these trajectories clustered in the age group of 13–15 years.

**Figure 2:**
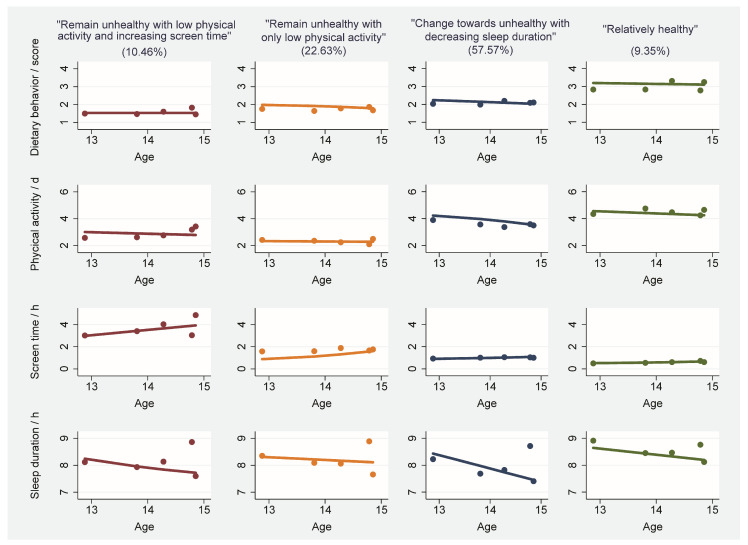
Multi-trajectory analysis of lifestyle behaviors among middle school students.

Figure 2 displays the estimated mean levels across four waves for each trajectory group in the four health-related behaviors. Students in the “remain unhealthy with low PA and increasing ST” group primarily exhibited increasing ST (2.5 h/day to 4 h/day) and lower levels of PA (approximately 3 days/week) with age. Additionally, they demonstrated other unhealthy behaviors; for example, their dietary behavior score was the lowest (1–2 scores) and their sleep duration decreased (8.2 h/day to 7.6 h/day) with age. Students in the “remain unhealthy with only low PA” group mainly showed the lowest PA (approximately 2 days/week) with age, lower levels of the dietary behavior score (approximately 2 scores), marginally increased ST (1 hour/day to 2 h/day), and slightly decreased sleep duration (8.2 h/day to 8 h/day) with age. Students in the “change towards unhealthy with decreasing sleep duration” group had a moderate dietary behavior score (approximately 2.2 scores) and dramatically decreased sleep duration (8.4 h/day to 7.5 h/day). They also had relatively lower levels of ST (approximately 1 hour/day) and slightly decreased PA (4 days/week to 3.8 days/week) with age. Additionally, compared with the other groups, students in the “relatively healthy” group displayed consistently high levels of the dietary behavior score (approximately 3.2 scores) and PA (approximately 4 days/week) with age, while exhibiting stable low ST (approximately 1.2 h/day) and relatively adequate sleep duration (approximately 8–9 h/day) with age. Overall, the changes in the four trajectories from the “unhealthy with low PA and increasing ST” to the “relatively healthy” groups exhibited a trend in transitioning from unhealthy to healthy among middle school students.

Table 1 presents the differences in sociological and demographic characteristics of middle school students across different lifestyle trajectories. The results indicated significant differences in the children’s sex and age among the different trajectory groups (*p* < 0.05). Additionally, the healthier the students’ lifestyle trajectories, the higher the perceived economic level of their families and the higher the educational level of their mothers.

### 3.2. Associations between Students’ Lifestyle Trajectories and Later BP

**Figure 3:**
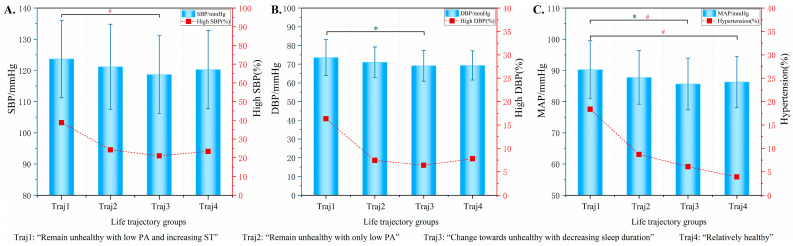
Comparison of BP in different lifestyle trajectory groups among middle school students. (**A**) Comparison of students’ SBP values and prevalence of high SBP in different lifestyle trajectory groups. (**B**) Comparison of students’ DBP values and prevalence of high DBP in different lifestyle trajectory groups. (**C**) Comparison of students’ MAP values and prevalence of hypertension in different lifestyle trajectory groups. * Comparisons of students’ BP values among lifestyle trajectory groups using the LSD test; the blue asterisk (*) designates significance at *p* < 0.05. # Comparisons of students’ HBP prevalence among lifestyle trajectory groups using the Bonferroni method; the red pound sign (#) designates significance at *p* < 0.05. Abbreviations: SBP, systolic blood pressure; DBP, diastolic blood pressure; MAP, mean arterial pressure; PA: physical activity; ST: screen time.

The BP values and prevalence rates of high SBP, DBP, and hypertension for the four lifestyle trajectories among middle school students are shown in Figure 3. The two-way comparison results revealed that students in the “change towards unhealthy with decreasing sleep duration” group had lower DBP and MAP values compared with those in the “remain unhealthy with low PA and increasing ST” group (*p* < 0.05). In addition, students in the “change towards unhealthy with decreasing sleep duration” group had lower prevalence rates of high DBP and hypertension compared with those in the “remain unhealthy with low PA and increasing ST” group (*p* < 0.05). Furthermore, students in the “relatively healthy” group had a lower prevalence rate of hypertension compared with those in the “remain unhealthy with low PA and increasing ST” group (*p* < 0.05).

As shown in Table 2, compared with students in the “relatively healthy” group, students in the “remain unhealthy with low PA and increasing ST” group had higher DBP (β = 3.49 [95% CI 0.55–6.44] mmHg) and MAP (β = 3.19 [95% CI 0.37 to 6.01] mmHg). It was also found that with the transition of lifestyle trajectory levels from the “relatively healthy” group to the “remain unhealthy with low PA and increasing ST” group, the students’ DBP and MAP showed a gradually increasing trend (*p* for trend < 0.05).

**Figure 4:**
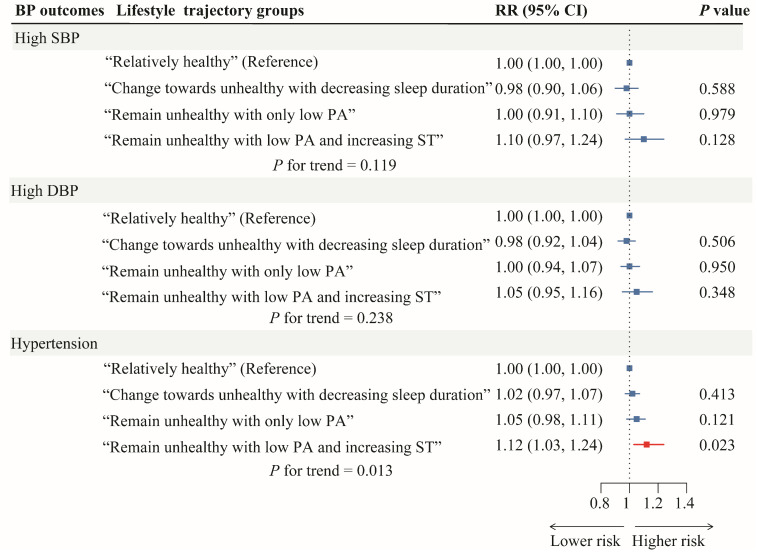
Adjusted risk ratios with 95% confidence intervals for associations between students’ lifestyle trajectories and BP outcomes. All models were adjusted for the age, sex, BMI, and self-perceived family income of the children and maternal education using modified Poisson regression. The error lines indicate 95%CIs, and the red error lines represent statistical significance for the prevalence of hypertension using the “relatively healthy” group as the reference category. Abbreviation: BP, blood pressure; DBP, diastolic blood pressure; MAP, mean arterial pressure; PA: physical activity; SBP, systolic blood pressure; RR, risk ratio; ST, screen time.

The adjusted associations between the students’ lifestyle trajectories and the risk of later high SBP, high DBP, and hypertension are depicted in Figure 4. After adjusting for important confounders, students in the “remain unhealthy with low PA and increasing ST” group (compared with the “relatively healthy” group) were associated with an increased risk of later hypertension (RR = 1.12; 95% CI, 1.03 to 1.24), and the trend in *p* from the “relatively healthy” group to the “remain unhealthy with low PA and increasing ST” trajectory group was 0.013.

### 3.3. Sensitivity Analyses

Considering that smoking and drinking alcohol may significantly affect the students’ BP, these two variables were included in the original model as additional confounding factors. We reanalyzed the relationship between the students’ lifestyle behavior trajectories and BP.

The results showed that the smoking and alcohol drinking rates among students were 3.85% and 21.95%, respectively. Additionally, compared with the “relatively healthy” group, students in the “remain unhealthy with low PA and increasing ST” trajectory group were associated with an increased risk of later hypertension (RR = 1.13; 95% CI, 1.02 to 1.25) (Supplementary Tables S3 and S4). The sensitivity analysis results were consistent with the primary analysis.

## 4. Discussion

In this study, we explored the joint lifestyle behaviors trajectory patterns of the dietary behavior score, PA, ST, and sleep duration, as well as associations with later BP, in middle school students. We identified four distinct lifestyle trajectories. More than half (57.57%) of the students were categorized in the “change towards unhealthy with decreasing sleep duration” group, only 9.35% of the students remained in the “relatively healthy” group, whereas 22.63% and 10.46% of the students belonged to the “remain unhealthy with only low PA” and “remain unhealthy with low PA and increasing ST” groups. The students in the “remain unhealthy with low PA and increasing ST” trajectory group were significantly associated with higher SBP and MAP, as well as an increased risk of hypertension, compared with those in the “relatively healthy” trajectory group. The above results confirmed our research hypothesis.

The unique nature of lifestyle behaviors in children and adolescents has been the focus of numerous studies. Previous research has examined individual lifestyle factors in isolation, such as diet, PA, ST, and sleep. However, our study stands out by identifying and analyzing four joint lifestyle behavior trajectories, which provides a more comprehensive understanding of how these behaviors interact and evolve over time. Our study found that more than half (57.57%) of the students were categorized in the “change towards unhealthy with decreasing sleep duration” group, who exhibited decreasing sleep duration and lower dietary behavior scores. These results are similar to those from a school-based cross-sectional study of Chinese adolescents, where the majority of adolescents (63.7%) had sleep deprivation and poor eating habits [34]. Also, several prospective cohort studies explored the longitudinal trajectories of various health behaviors in children and adolescents. For instance, Zhang et al. [17]. conducted a trajectory analysis on four-year longitudinal data of 1974 children aged 7–9 in Anhui, China, examining behaviors such as ST, PA, sleep duration, and sweetened beverages. They found that fewer than 40% of the Chinese children were classified as having a healthy lifestyle, while 20% were identified as maintaining a persistently unhealthy lifestyle, characterized by consistently high ST and sweetened beverage intake, along with a significant reduction in sleep duration (less than 8 h per day). Differing from that study, only 9.35% of the individuals in our study fell into the “relatively healthy” lifestyle trajectory; these individuals had consistently high levels of dietary behavior scores, PA, stable low ST, and relatively adequate sleep duration. However, it is worth noting that the Zhang et al. study focused on elementary school students, and it is unclear whether this pattern of health continues into middle school. Additionally, the GUSTO study analyzed three follow-up datasets of children aged 2–8, focusing on diet, PA, ST, and sleep. It ultimately identified the following joint lifestyle trajectories: consistently healthy, mixed pattern, and unhealthy [16]. They found that the majority of children (71%) followed a mixed pattern trajectory, characterized by low PA, moderate ST, and low consumption of fruits, vegetables, and discretionary foods. The above studies suggested that there may be different patterns in trajectory clustering for early childhood and adolescent lifestyle behaviors.

The association between lifestyle behaviors and BP in children has been well documented. For instance, studies have shown that poor dietary habits, such as high consumption of sugary drinks [10] and ultra-processed foods [35,36], were linked to elevated BP. Similarly, inadequate PA [13,37], excessive ST [15,38], and insufficient sleep [39] have been associated with higher BP levels. Our study demonstrated that the remaining unhealthy lifestyle trajectory with low PA and increasing ST was associated with an increasing risk of hypertension in students, whereas the unhealthy lifestyle trajectory with only low PA was not. Previous studies have demonstrated that PA has a high level of evidence for its impact on the BP of children and adolescents, while the evidence for screen time is relatively lower [24]. Our research found that consistently insufficient PA, along with increasing ST, among middle school students may be associated with elevated BP. That is, the combined effect of PA and ST behaviors may appear to have a synergistic impact on BP regulation, suggesting that interventions targeting multiple behaviors simultaneously could yield more substantial health benefits compared with single-behavior interventions. It is noteworthy that the unhealthy groups, including the “remain unhealthy with only low PA” and “change towards unhealthy with decreasing sleep duration” groups, also exhibit unhealthy lifestyles but are not associated with students’ elevated BP. This may be due to the short follow-up period and the possibility that a single unhealthy lifestyle may not be sufficient to affect subsequent BP. However, over time, these individuals may develop cardiovascular and metabolic abnormalities in late adolescence or adulthood.

Furthermore, aligned with previous studies [40,41], we discovered that students from wealthier families and with more educated mothers were more likely to develop healthy behavior and lifestyle patterns, which indicates that sociodemographic factors are pivotal in influencing students’ behavioral lifestyles. Consequently, social policy interventions may be an effective tool in enhancing students’ behavioral lifestyles. Currently, research on the joint trajectories of lifestyle behaviors and BP in children and adolescents is sparse. Only one study indicated that among children aged 2–8 years, maintaining a healthy lifestyle trajectory—characterized by high intakes of fruit and vegetables, low ST, high PA, outdoor play, and participation in organized PA—was associated with a reduced risk of developing prehypertension. Our findings were similar; however, this study utilized a larger sample size and focused on middle school students, thereby providing additional evidence for this field.

Our study also has significant public health and clinical implications. From a public health perspective, our study highlighted the need for integrated lifestyle interventions that promote healthy behaviors across multiple domains. School-based programs and family-centered initiatives that promote balanced diets, regular physical activity, adequate sleep, and limited screen time are crucial in mitigating the risk of hypertension in children. Clinically, our findings suggest that healthcare providers should adopt a holistic approach when advising students and their families on lifestyle changes. By addressing multiple lifestyle factors simultaneously, particularly focusing on students with low PA and excessive ST, hypertension prevention can be more effectively achieved.

A major strength of our study is the use of group-based multi-trajectory modeling, which allowed us to capture the complexity and interdependence of lifestyle behaviors over time. Additionally, the longitudinal design enhanced the robustness and generalizability of our findings.

However, our study has some limitations. First, the participants in this study were middle school students aged 12–18. The lifestyle behavior data were collected through self-reports from the students, which may introduce reporting bias. Second, this study did not consider unmeasured covariates, such as genetic and prenatal factors, which may affect the accuracy of causal inferences. Third, the study population was confined to a specific district in Shanghai, which may limit the extrapolation of our findings to other regions or populations. Future studies should be devoted to duplicating these findings in different populations and investigating potential mechanisms between lifestyle behaviors and BP.

## 5. Conclusions

In conclusion, our study identified four distinct lifestyle trajectories among middle school students and demonstrated that students maintaining an unhealthy lifestyle trajectory characterized by low PA and increasing ST were associated with elevated BP and an increased risk of hypertension. These findings suggest that holistic lifestyle interventions may help mitigate the risk of hypertension in children.

## Figures and Tables

**Table 1:** Characteristics of lifestyle trajectories among school-aged children [Mean ± S/median [IQR]/N (%)].

Characteristic	Overall	“Remain Unhealthy with Low PA and Increasing ST”	“Remain Unhealthy with Only Low PA”	“Change towards Unhealthy with Decreasing Sleep Duration”	“Relatively Healthy”	*p*
**N**	1348	141 (10.46)	305 (22.63)	776 (57.57)	126 (9.35)	
**Sex (%)**						
Boys	795 (58.98)	103 (73.05)	202 (66.23)	417 (53.74)	73 (57.94)	<0.001
Girls	553 (41.02)	38 (26.95)	103 (33.77)	359 (46.26)	53 (42.06)	
**Age (median [IQR])**	14.36 [13.30, 17.28]	13.69 [12.69, 17.20]	17.07 [14.14, 17.58]	13.96 [12.88, 14.99]	14.48 [13.48, 17.09]	<0.001
**Self-perceived family income (%)**						
Fine	506 (37.54)	27 (19.15)	114 (37.38)	308 (39.69)	57 (45.24)	<0.001
Poor	842 (62.46)	114 (80.85)	191 (62.62)	468 (60.31)	69 (54.76)	
**Maternal education (%)**						
Junior high school or below	323 (23.96)	74 (52.48)	85 (27.87)	143 (18.43)	21 (16.67)	<0.001
High school	251 (18.62)	23 (16.31)	53 (17.38)	155 (19.97)	20 (15.87)	
College degree or above	774 (57.42)	44 (31.21)	167 (54.75)	478 (61.60)	85 (67.46)	
**n**	615	49 (8.01)	161 (26.31)	328 (53.59)	77 (12.68)	
**BMI (median [IQR])**	20.36 [17.88, 24.07]	19.70 [17.40, 23.85]	20.86 [18.36, 24.24]	20.07 [17.70, 23.89]	20.99 [18.44, 23.74]	0.172
**SBP (mean [SD])**	119.93 (12.86)	123.67 (12.46)	121.18 (13.63)	118.67 (12.47)	120.29 (12.58)	0.031
**DBP (mean [SD])**	70.01 (8.36)	73.51 (9.59)	71.04 (8.17)	69.15 (8.23)	69.30 (7.80)	0.002
**MAP (mean [SD])**	86.65 (8.55)	90.23 (9.32)	87.76 (8.58)	85.66 (8.31)	86.29 (8.25)	0.001
**High SBP (%)**						
Normal	470 (76.42)	30 (61.22)	122 (75.78)	259 (78.96)	59 (76.62)	0.060
High SBP	145 (23.58)	19 (38.78)	39 (24.22)	69 (21.04)	18 (23.38)	
**High DBP (%)**						
Normal	568 (92.36)	41 (83.67)	149 (92.55)	307 (93.60)	71 (92.21)	0.110
High DBP	47 (7.64)	8 (16.33)	12 (7.45)	21 (6.40)	6 (7.79)	
**Hypertension (%)**						
Normal	569 (92.52)	40 (81.63)	147 (91.30)	308 (93.90)	74 (96.10)	0.010
Hypertension	46 (7.48)	9 (18.37)	14 (8.70)	20 (6.10)	3 (3.90)	

Data are presented as mean ± standard deviation/median [interquartile range] or as number (frequency). “N” represents the sample size included in the trajectory model, consisting of students who had more than three waves of lifestyle monitoring data in 2019–2023; “n” represents the final sample size, which includes students who had more than three waves of lifestyle monitoring data in 2019–2023 and BP measurements in 2023. Abbreviations: BMI, body mass index; BP, blood pressure; SBP, systolic blood pressure; DBP, diastolic blood pressure; IQR: interquartile range; MAP, mean arterial pressure; PA: physical activity; SD: standard deviation; ST: screen time.

**Table 2:** Associations between lifestyle trajectory and BP among middle school students.

Lifestyle Trajectory Groups	SBP		DBP		MAP	
	β (95% CI)	*p*	β (95% CI)	*p*	β (95% CI)	*p*
“Relatively healthy”	Reference		Reference		Reference	
“Change towards unhealthy with decreasing sleep duration”	−0.68 (−3.41, 2.06)	0.628	−0.05 (−2.07, 1.97)	0.964	−0.26 (−2.19, 1.68)	0.795
“Remain unhealthy with only low PA”	0.02 (−2.98, 3.03)	0.987	1.32 (−0.90, 3.54)	0.243	0.89 (−1.24, 3.01)	0.412
“Remain unhealthy with low PA and increasing ST”	2.58 (−1.41, 6.57)	0.205	**3.49 (0.55, 6.44)**	**0.020**	**3.19 (0.37, 6.01)**	**0.027**
*p* for trend	0.200		**0.006**		**0.012**	

All models were adjusted for the age, sex, BMI, and self-perceived family income of the children and maternal education using the GLM model. The bolded effect sizes indicate statistical significance (*p* < 0.05). Abbreviations: SBP, systolic blood pressure; DBP, diastolic blood pressure; MAP, mean arterial pressure; PA: physical activity; ST: screen time.

## Data Availability

The data and analytic code described in this manuscript are available from the corresponding author upon reasonable request. The data are not publicly available due to participant privacy.

## Supplementary Materials

The following supporting information can be downloaded at https://www.mdpi.com/article/10.3390/nu16172994/s1, Table S1: Model fit statistic parameters of the group-based multi-trajectory modeling; Table S2: Descriptive analysis of lifestyle measures across five waves among school-aged children in 2019–2023; Table S3: Sensitivity analysis of associations between lifestyle trajectory and BP among middle school students, additionally adjusted the child smoking, and child alcohol-drinking; Table S4: Sensitivity analysis of adjusted risks ratios with 95% confidence intervals for associations between students’ lifestyle trajectory and BP outcomes, additionally adjusted the child smoking, and child alcohol-drinking.
