# Supplementary material for: Joint Trajectories of Lifestyle Indicators and Their Associations with Blood Pressure among Chinese Middle School Students
Source: Nutrients. 2024 Sep 5;16(17):2994. doi: 10.3390/nu16172994 (PMC11396788; doi:10.3390/nu16172994)
Supplement: Supplementary file 1 [file nutrients-16-02994-s001.zip › nutrients-3181496-supplementary.pdf]

# Joint trajectories of lifestyle indicators and their associations with blood pressure among Chinese middle school students

**Table S1**. Model fit statistics parameter of the group-based multi-trajectory modeling.

**Table S2.** Descriptive Analysis of Lifestyle Measures across Five Waves among middle school students in 2019-2023.

**Table S3.** Sensitivity analysis of associations between lifestyle trajectory and BP among middle school students, additionally adjusted the child smoking, and child alcohol-drinking.

**Table S4.** Sensitivity analysis of adjusted risks ratios with 95% confidence intervals for associations between students' lifestyle trajectory and BP outcomes, additionally adjusted the child smoking, and child alcohol-drinking.

Table S1. Model fit statistics parameter of the group-based multi-trajectory modeling.

| Factors | Model | Average posterior probability% | Proportions per class% | BIC | AIC | Odds of correct classification | Entropy |
|---|---|---|---|---|---|---|---|
| Diet | 4_3031 | 0.85/0.88/0.80/0.80 | 59.75/9.46/19.21/11.58 | -30100.49 | -29894.1 | 4.22/61.22/12.84/35.63 | 0.713 |
| Physical activity | 4_3111 | | | | | | |
| Screen time | 4_2332 | | | | | | |
| Sleep time | 4_3211 | | | | | | |

Table S2. Descriptive Analysis of Lifestyle Measures across Five Waves among middle school students in 2019-2023.

| Lifestyle behavior indicators | 2019 | 2020 | 2021 | 2022 | 2023 |
|---|---|---|---|---|---|
| N | 1785 | 1924 | 1968 | 1927 | 1944 |
| Dietary behavior score (number of ideal metrics) | | | | | |
| Median (median [IQR]) | 2.00 [1.00, 2.00] | 4.00 [3.00, 4.00] | 2.00 [2.00, 3.00] | 2.00 [2.00, 3.00] | 2.00 [2.00, 2.00] |
| Mean (SD) | 1.88 (0.78) | 3.76 (0.74) | 2.16 (0.94) | 2.09 (0.96) | 2.11 (0.81) |
| $F^{a}$ | | | 1551.16 | | |
| *P* value | | | <0.001 | | |
| Physical activity (＞1 h/d, d/w) | | | | | |
| Median (median [IQR]) | 3.00 [1.00, 5.00] | 3.00 [2.00, 5.00] | 3.00 [2.00, 5.00] | 3.00 [2.00, 5.00] | 3.00 [2.00, 5.00] |
| Mean (SD) | 3.13 (2.22) | 3.22 (2.19) | 3.28 (2.18) | 3.48 (2.23) | 3.24 (2.21) |
| $\chi^{2\ b}$ | | | 26.67 | | |
| *P* value | | | <0.001 | | |
| Screen time (hours/d) | | | | | |
| Median (median [IQR]) | 1.00 [0.50, 2.17] | 1.00 [0.50, 2.00] | 1.00 [0.33, 2.00] | 1.00 [0.17, 2.00] | 1.00 [0.17, 2.08] |
| Mean (SD) | 1.58 (1.65) | 1.50 (1.75) | 1.53 (1.89) | 1.52 (2.24) | 1.40 (1.46) |
| $\chi^{2\ b}$ | | | 35.04 | | |
| *P* value | | | <0.001 | | |
| Sleep duration (hours) | | | | | |
| Median (median [IQR]) | 8.00 [7.00, 9.00] | 8.00 [6.70, 8.50] | 8.00 [7.00, 9.00] | 8.00 [7.00, 9.00] | 9.00 [8.00,10.00] |
| Mean (SD) | 7.86 (1.34) | 7.72 (1.43) | 8.13 (1.39) | 7.83 (1.64) | 8.95 (1.24) |
| $F^{a}$ | | | 233.89 | | |
| *P* value | | | <0.001 | | |

[a] denotes that the variable is normally distributed and was compared using ANOVA;

[b] denotes that the variable is not normally distributed and was compared using nonparametric test.

Table S3. Sensitivity analysis of associations between lifestyle trajectory and BP among middle school students, additionally adjusted the child smoking, and child alcohol-drinking.

| Lifestyle trajectory groups | SBP | | DBP | | MAP | |
|---|---|---|---|---|---|---|
| | β (95% CI) | *P* | β (95% CI) | *P* | β (95% CI) | *P* |
| "Relatively Healthy" | Reference | | Reference | | Reference | |
| "Change towards unhealthy with decreasing sleep duration" | -0.72 (-3.45,2.02) | 0.606 | 0.01 (-2.02,2.03) | 0.994 | -0.23 (-2.17,1.7) | 0.812 |
| "Remain unhealthy with only low PA" | 2.61 (-1.4,6.62) | 0.201 | 1.46 (-0.76,3.68) | 0.197 | 0.98 (-1.14,3.11) | 0.364 |
| "Remain unhealthy with low PA and increasing ST" | 0.03 (-2.98,3.03) | 0.986 | **3.62 (0.66,6.59)** | **0.017** | **3.29 (0.45,6.12)** | **0.023** |
| *P* for trend | 0.195 | | **0.004** | | **0.01** | |

Adjusted for family income, maternal education, child age, child sex, child BMI, child smoking, and child alcohol-drinking.

The bolded effect sizes indicate statistical significance (*P*<0.05).

Table S4. Sensitivity analysis of adjusted risks ratios with 95% confidence intervals for associations between students' lifestyle trajectory and BP outcomes, additionally adjusted the child smoking, and child alcohol-drinking.

| Lifestyle Multi-trajectories | High SBP | | High DBP | | Hypertension | |
|---|---|---|---|---|---|---|
| | RR (95% CI) | *P* | RR (95% CI) | *P* | RR (95% CI) | *P* |
| "Relatively Healthy" | Reference | | Reference | | Reference | |
| "Change towards unhealthy with decreasing sleep duration" | 0.86 (0.54, 1.37) | 0.525 | 0.73 (0.30, 1.80) | 0.515 | 1.02 (0.974, 1.08) | 0.367 |
| "Remain unhealthy with only low PA" | 1.00 (0.62, 1.66) | 0.957 | 1.07 (0.41, 2.78) | 0.885 | 1.05 (0.99, 1.11) | 0.116 |
| "Remain unhealthy with low PA and increasing ST" | 1.40 (0.80, 2.44) | 0.233 | 1.52 (0.54, 4.25) | 0.423 | **1.13 (1.02, 1.25)** | **0.021** |
| *P* for trend | 0.144 | | 0.209 | | **0.014** | |

Adjusted for family income, maternal education, child age, child sex, child BMI, child smoking, and child alcohol-drinking.

The bolded effect sizes indicate statistical significance ($P$<0.05).
